# “La Perla Del Mar”: A Case Report on Subcutaneous Penile Implants

**DOI:** 10.7759/cureus.37155

**Published:** 2023-04-05

**Authors:** Juan C Ramirez, Praveen D Wickremasinghe, Luis X Mayol-Velez, Guillermo Izquierdo-Pretel

**Affiliations:** 1 Internal Medicine, Herbert Wertheim College of Medicine, Florida International University, Miami, USA

**Keywords:** artificial penile nodules, genital modification, sexual education, subcutaneous penile implant, penile implants

## Abstract

The purpose of this report is to alert and inform the medical community about the presence and practice of subcutaneous penile implants (SPIs), which are used with the intent of increasing sexual pleasure. This case aspires to deflect plausible misconceptions in the specific populations who use the SPIs. This case study was performed in January 2023 at a tertiary care center in Miami, Florida. A 61-year-old Cuban male admitted for a routine hernia repair with an incidental finding of a benign SPI was interviewed and examined; an extended collection of historical information regarding the patient’s penile implant was ascertained. The patient stated that there was a tradition among the men and adolescent individuals living along coastal cities/towns of Cuba such as Havana and Matanzas who would elect to have pieces of stones or gems or any solid objects shaped and molded into round objects that are used for the intent of increasing sexual pleasure. The patient referred to the implant as “La Perla Del Mar,” which translates directly into “Pearl of the Sea.” Upon visualization of the nodule on examination, a differential diagnosis may include infection (such as syphilis), granulomas, sarcoidosis, dermatofibroma, epithelial inclusion cyst, or malignancy. However, an appropriate workup informed us about the penile implant. Clinicians should employ caution in investigating a penile nodule by taking a detailed social and sexual history and physical exam from the patient if possible. This case and the literature review cited to bolster the notion of a lack of chronic symptoms due to the inserted objects. Several provocations for the implantation of an artificial penile nodule, in this case, maybe extrapolated, such as the desire for a prospective partner’s pleasure/displeasure, group identification, or masculine embodiment. The main takeaways from this case report are the considerations that should be taken in the older Caribbean population for patients with the “Perla Del Mar” implantation and bolstering the notion of complete sexual education for clinicians regarding specific populations to enhance patient care.

## Introduction

Penile modification has long been a tradition in some regions of the world, and the stigma of external modification has evolved over time in the cultural contexts they hold. The earliest recorded instances of penile modifications were mentioned as far back as in the Kama Sutra as a method to enhance sexual satisfaction [1]. In Western society, this form of modification, subcutaneous penile implants (SPIs), had been an almost unheard-of tradition. SPIs are more common in Southeast Asian culture due to the belief in the augmentation of sexual pleasure and arousal, and this trend has been slowly growing in popularity in Western culture [1, 2]. From an anthropological view, modification of one's genitals was originally thought to be a rite of passage [3], which corresponds to the patient's beliefs in this practice. In an examination of the societies that still partake in some form of it, it is done at the behest of the individual for cultural, sexual, or aesthetic purposes. For instance, sailors from the Philippines engage in the pearling practice, referring to them as "bolitas," and this was used for both sexual purposes, attempting to attract sex workers, as well as a form of identification between themselves and other sailors from different countries [4]. In Western society, body modification is slowly becoming more welcome, and trends involving penile external modification are rising. As noted by Fischer N et al. in their conclusions of artificial penile nodules, our Western society is always looking for new and exotic practices to follow to augment aesthetics or exhibit an increased level of their sexuality [4, 5]. It was deemed necessary to both inform and educate the medical community of the West at large about this practice, which has always been ongoing but is slowly becoming more frequent. Upon review of the existing literature, SPI is a practice that appears to have originated and is more prevalent in Southeast Asian culture, including Thailand and Indonesia, where migrant workers would carry the practice to neighboring countries and lands [6]. The exact genesis of the practice is unknown, but the earliest recordings of it are from China, which shows it was imported from Southeast Asia around the early 1400s. The objects used as SPIs have been described in the literature as pieces of hard pearls, glass beads, hard objects, sharpened dominoes, and cut/shaped pieces of ivory, gems, stones, bullets, plastic, and even gold [2, 6]. The number of beads used as SPIs varies by individual, depending on personal choice and the person's socioeconomic status. Past reports detailed that the individuals who bear SPIs include sailors, soldiers, prisoners, and sex workers [7].

Hull TH and Budiharsana M (2001) wrote about the cultural views of penile modifications and the different types of them, including SPIs, and how that dependent on the material used, it can bestow upon the individual luck or possess attributes that can enhance sexual potency [7]. A form of SPI has also been documented for use in Japan by the criminal organization, the Yakuza. They are noted to not only use full body tattooing (irezumi), but also the implantation of pearlings. What is unique about this practice is that variation of SPI is that for members, they would insert the pearlings under the foreskin for each year of confinement in prison with the intention of identification of membership in the organization as well as the intention to "recompensate" the female sexual partners with increased sexual pleasure and satisfaction for the absence of the male individual [8]. In some cultures in Asia, the SPI nodules reveal the status of the bearer in their groups, and there is a belief that not only will sexual satisfaction be increased, but the individual will gain some luck from it [9]. Most of the reported uses have been for the augmentation of pleasure from sexual activity. However, Hull TH and Budiharsana M did report that some SPI individuals have undergone the procedure to inflict pain on partners during sexual intercourse. Other pieces of literature report that women injured by SPIs have sustained genital trauma, including lacerations and abrasions. [7]. Given the rise in popularity, it is important for clinicians to learn about and be aware of this practice and, dependent on a patient's present symptomatology, whether this should be a cause for concern or just an incidental finding on a physical exam. It is essential to identify the practice as something that is occurring and not something that is misinterpreted as a possible pathology that would then go on to be managed medically unnecessarily. In this article, we present a case report for a patient with an asymptomatic SPI. We describe the medical and social history associated with the patient's history with his SPI, which he referred to as "La Perla."

## Case presentation

**Figure 1:**
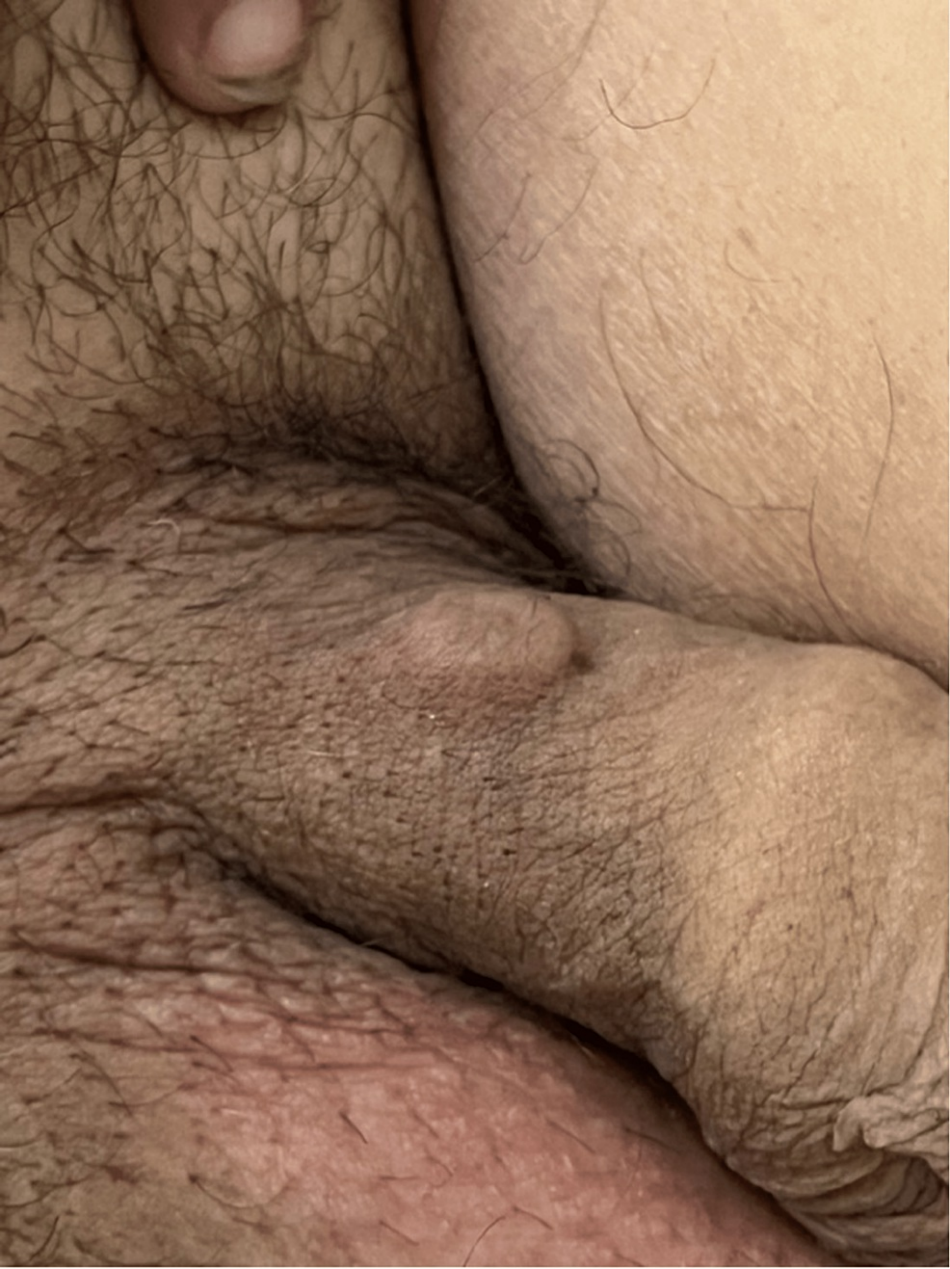
"La Perla Del Mar." Approximately 1 cm subcutaneous penile implant forming a nodule, about 1 inch above the base of the penis on the dorsal aspect. No signs of overt erythema, warmth, swelling, or tenderness were noted on the physical exam. A hyperpigmented mark on the distal aspect of the nodule is present, most likely indicating the incision mark from when it was first made.

**Figure 2:**
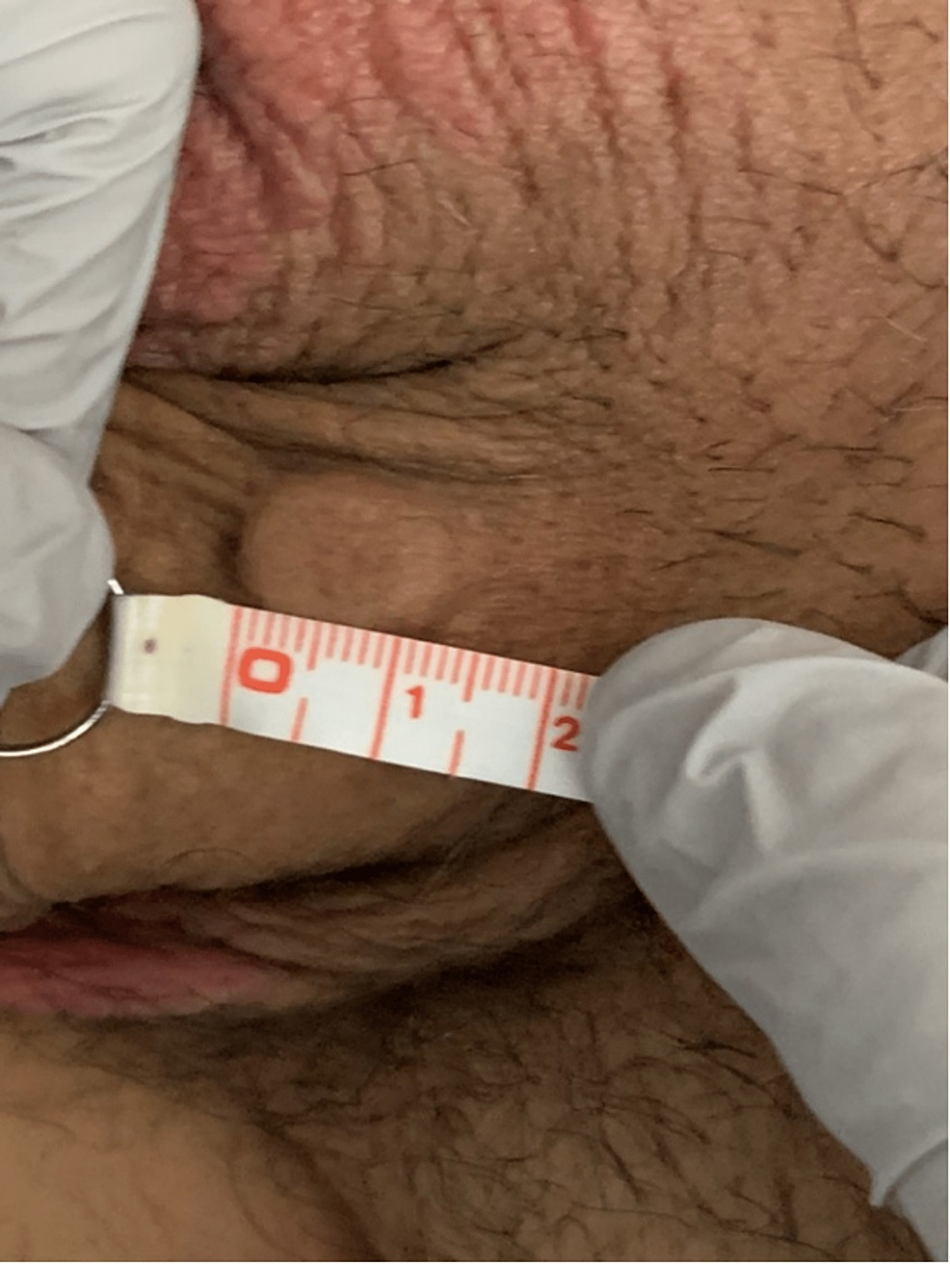
Dorsal view of "La Perla Del Mar." Raised nodule was 1.2 cm in length.

**Figure 3:**
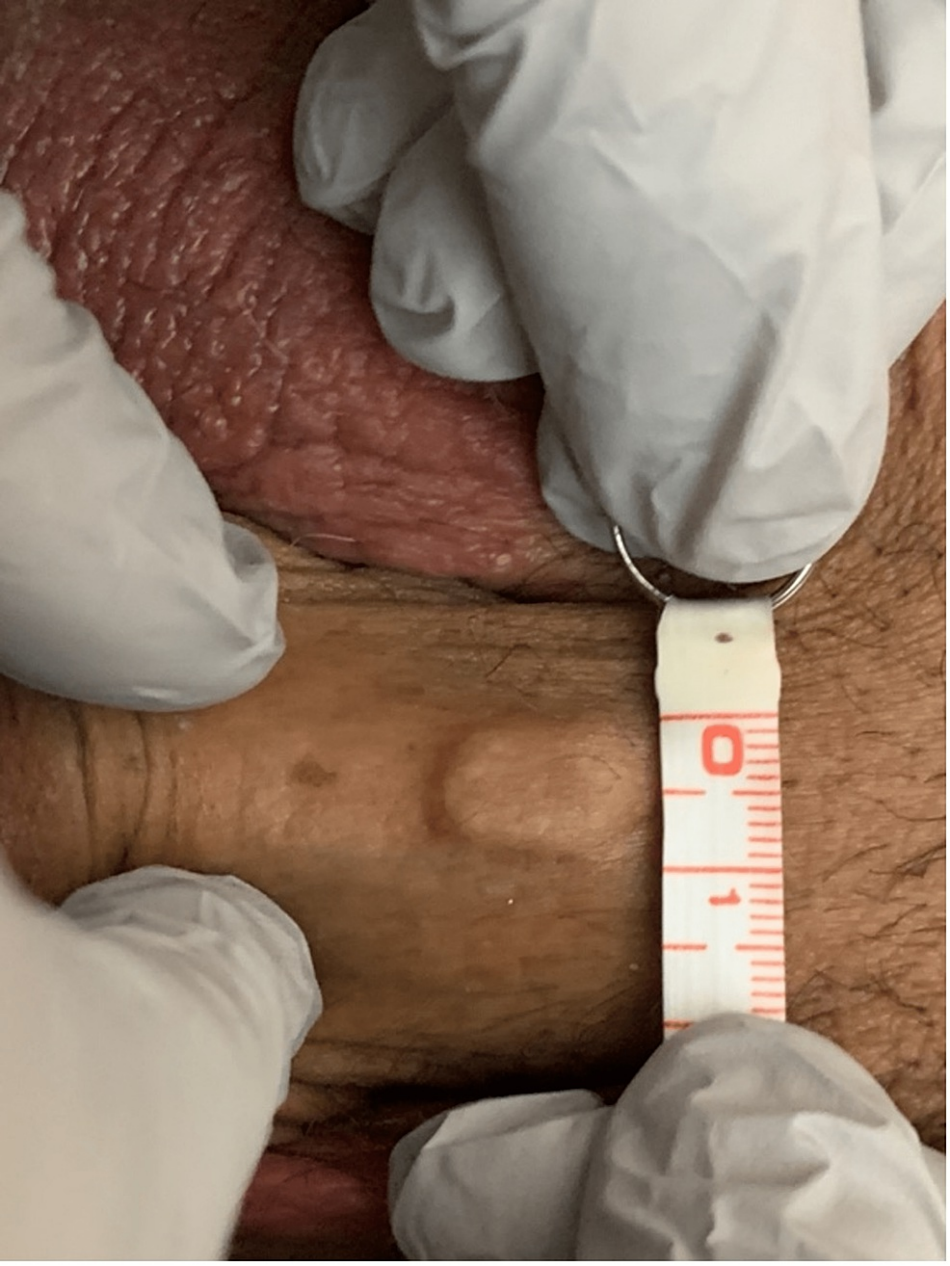
Measurements of "La Perla Del Mar." Width of the nodule measuring 0.9 cm.

This 61-year-old patient was admitted to our service at a tertiary care center in Miami, Florida, for an initial consult of a right-sided hernia that was discovered to be, upon workup, an incarcerated hernia. During the patient's hospitalizations, a few complications occurred, including the induction of atrial fibrillation during surgery and what appeared to be a failure of initial surgical intervention and a re-herniation of bowels into the scrotal sac that caused exquisite tenderness and was partially relieved with opioids. During the evaluation of the patient, there was an incidental finding on the physical exam of a 1.2 x 0.9 cm raised nodule that was non-tender, non-erythematous, and lacked any other signs of an acute infectious or inflammatory process (Figures 1-3). The patient was at first apprehensive about the initial inquiry into what appeared at first glance to be a pathogenic lesion but eventually became open to answering questions about the nodule and its purpose. 

The patient reported that in his native Cuba, it was a tradition of adolescent males (around the age of 15, which is the same age he himself was at the time) who live by the coast and in other cities/towns close by to have this SPI placed with the intent to enhance sexual pleasure for both him and his sexual partners (the patient self-reported as heterosexual during the interview). The logic is that the SPI is placed approximately an inch above the dorsal base of the shaft of the penis with the expectation that it travels up and down and bidirectionally during intercourse and is meant to rub against the female clitoris. The patient reports that he had the procedure done in a non-hospital setting by a nurse. He reports that he selected a stone he found and had it soaked in "90-proof alcohol" for approximately three days prior to implantation. During the procedure, he mentioned using an alcohol-based product acting as an anti-septic. A surgical incision was made to penile skin using an object with a sharp point. The implant was inserted under the skin through the incision towards the dorsum of the shaft of the penis, consistent with how the procedure has been done recently [5]. He reports he took prophylactic antibiotics (penicillin-based) for infection prevention. The patient reports that he did not develop any infection from the procedure in the days and weeks following the implantation and that he was instructed to ensure proper placement of the SPI by pulling up and down on the penis to confirm the mobility of the implant. When asked about penile concerns, he denies any history of erectile dysfunction or difficulty urinating or ejaculating. He denies any prior history of sexually transmitted infections (STIs) before or after placement. He denies ever having it needed to be removed for any reason or replaced at any time. When asked more about his reasoning and understanding of the cultural practice, the patient states that he had heard this practice originated in Asia, where a medical doctor would implant stones or gems into the base of the penis to serve individuals who would frequent "prostitution houses." He reports he was in the military service of Cuba when he had the SPI placed, given that he was moving across different cities, towns, and countries and was planning to engage in sexual activity with multiple partners in these different locations. He denies that he ever injured his sexual partners with the SPI and denies that he ever had the intent to hurt people with it. The patient admits to engaging in risky behaviors such as having multiple sexual partners, and he denies any ever-using barrier contraception during sexual activity. He reported that he has had increased sexual pleasure as well as some of his female sexual partners. He recounted them, saying their pleasure had been increased because of the SPI. He did not report any instance of engaging in sexual activity with any partner before or after SPI placement.

## Discussion

This case report describes the instance of a Cuban gentleman with an incidental finding of an SPI that had been asymptomatic. What alerted the team to inquire further about this implant was the fact that this could have been potentially misinterpreted as a sign of pathology that would have required treatment. Upon first notice of a lesion or nodule on the penis, the initial thought of the team was that it could have been a potential STI, most likely a chancre, given its painless nature. Upon patient interview, we confirmed that this was not an STI. As mentioned above, most cases of SPIs have been documented in Asian cultures. However, given the fact that the patient, in this case report, is from Cuba, there are no pieces of academic literature currently written about this practice in Cuba or other Caribbean cultures, despite emphasis by the patient in the case report stating that this was a common practice in Cuba, throughout the island and not just along coastal cities as the tradition dictated and that he personally knew individuals living in Miami with SPIs as well. The patient never developed a systemic or localized infection due to the placement of the SPI. He has attributed it to the fact that he took antibiotics following the procedure and had alcohol used as an antiseptic for the stone, and irrigated himself prior to the incision. Kirkham CL et al. (2019) wrote a case report on seven patients that presented to the ED on the Southwest border of the United States with complications from different subcutaneous penile modifications [2]. All patients presented to the ED with pain; four out of the seven presented with signs of infection, four requiring removal of the foreign body [2]. The patient's reported history of the procedure he underwent matches what has been written by Murty OP (2008) in that the objects used for SPIs were spirit-cleaned or boiled prior to insertion [6]. The patient reports that he was given penicillin following the procedure and that alcohol was used as the antiseptic in a sterile field. This procedure was performed by whom he described as a nurse who most likely would have taken these precautions to strive for minimal risk of infection in an out-of-the-hospital setting.

Fischer N et al. continued to remark that these beads have no medical or epidemiological relevance [9]. The patient in the case admits to not having had any associated health difficulties; however, there is documented evidence of the complications that can arise with genital modifications and apparatuses, such as a reported instance of penile and scrotal incarceration secondary to prolonged use of a metal ring [10]. Kirkham CL et al. published a case series of different subcutaneous complications such as discomfort, infection, penile bleeding, or increased risk of infection, mostly attributed to the lack of unsterile conditions present during SPI placement. The anatomical placement of the SPI can also affect nearby structures, such as iatrogenic damage to the corpora, arteries, and nerves of the penis, and the majority of SPIs described in the case report by Murty OP were placed on the dorsum of the penis [6], in the exact location as our patient described earlier. As to the efficacy of whether SPIs work, this is all based on the opinion of the individual bearer, as there is a lack of available analyses or surveys that look at satisfaction with the implant. The patient reported satisfaction with "La Perla," and he recounted positive sexual experiences. Given the persistence of the practice in other cultures, despite the risk of infections or injuries, it can be inferred that there is at least not a significant net benefit or risk by most users for them to engage in the practice. Cultures and individuals that continue to engage in the practice should be made aware or encouraged to implement antiseptic techniques during SPI implementation so that at least the infection risks can be minimized.

## Conclusions

The main point of this case report is to inform clinicians of the practice of SPIs. Clinicians should be aware of the practice in order to avoid unnecessary intervention when they encounter patients with SPIs. This is a cosmetic procedure that has a variety of meanings depending on the individual's culture. Clinicians should be educated on the original meaning of what SPIs are for, how they are implanted, most often in a non-hospital or clinic setting, and what should be on the lookout for patients who present with potential signs and symptoms of infection due to SPIs. Consideration as well to the sexual partner of the SPI bearer is also essential to be considered, such as the complications that can arise from them, including dyspareunia, vaginal trauma, and a higher risk of STIs. Given the little knowledge and seemingly exotic nature of the practice of SPIs in Western society, it is important to make clinicians aware of the practice and educate them on how the procedure is done and the effects that can arise from the procedure. This case report could also serve as an opportunity to educate clinicians and the public on one facet of sexual health and sexual behaviors. It is valuable to understand the trends in the sexual behavior of the public and how these behaviors impact both the individual and the parties they are choosing to engage with. Misconceptions about the practices, such as who engages in them or what their use may entail regarding sexual behaviors, should be noted and cleared up for the public.
